# Identification and functional analysis of two P450 enzymes of *Gossypium hirsutum* involved in DMNT and TMTT biosynthesis

**DOI:** 10.1111/pbi.12797

**Published:** 2017-08-16

**Authors:** Danfeng Liu, Xinzheng Huang, Weixia Jing, Xingkui An, Qiang Zhang, Hong Zhang, Jingjiang Zhou, Yongjun Zhang, Yuyuan Guo

**Affiliations:** ^1^ State Key Laboratory for Biology of Plant Diseases and Insect Pests Institute of Plant Protection Chinese Academy of Agricultural Sciences Beijing China; ^2^ College of Plant Protection Shandong Agricultural University Tai'an Shandong China; ^3^ Department of Biological Chemistry and Crop Protection Rothamsted Research Harpenden UK

**Keywords:** DMNT and TMTT, *Gossypium hirsutum*, cytochrome P450, enzymatic activity assay, expression profile, EAG recording, behavioural response

## Abstract

The homoterpenes (*3E*)‐4,8‐dimethyl‐1,3,7‐nonatriene (DMNT) and (*E,E*)‐4,8,12‐trimethyl‐1,3,7,11‐tridecatetraene (TMTT) are major herbivore‐induced plant volatiles that can attract predatory or parasitic arthropods to protect injured plants from herbivore attack. In this study, DMNT and TMTT were confirmed to be emitted from cotton (*Gossypium hirsutum*) plants infested with chewing caterpillars or sucking bugs. Two CYP genes (*GhCYP82L1* and *GhCYP82L2*) involved in homoterpene biosynthesis in *G. hirsutum* were newly identified and characterized. Yeast recombinant expression and enzyme assays indicated that the two GhCYP82Ls are both responsible for the conversion of (*E*)‐nerolidol to DMNT and (*E,E*)‐geranyllinalool to TMTT. The two heterologously expressed proteins without cytochrome P450 reductase fail to convert the substrates to homoterpenes. Quantitative real‐time PCR (qPCR) analysis suggested that the two *GhCYP82L* genes were significantly up‐regulated in leaves and stems of *G. hirsutum* after herbivore attack. Subsequently, electroantennogram recordings showed that electroantennal responses of *Microplitis mediator* and *Peristenus spretus* to DMNT and TMTT were both dose dependent. Laboratory behavioural bioassays showed that females of both wasp species responded positively to DMNT and males and females of *M. mediator* could be attracted by TMTT. The results provide a better understanding of homoterpene biosynthesis in *G. hirsutum* and of the potential influence of homoterpenes on the behaviour of natural enemies, which lay a foundation to study genetically modified homoterpene biosynthesis and its possible application in agricultural pest control.

## Introduction

Plants promote self‐fitness against herbivore attack not only by producing toxins and repellents but also by emitting volatiles that attract natural enemies of herbivorous insects (Gols, 2014). It has been reported that herbivore‐induced plant volatiles (HIPV) play an important role in plant communication, functioning as airborne cues to induce defence in adjacent foliage or plants or to prime uninfected plant tissue for potentiated defence responses upon subsequent herbivore attack (Turlings and Ton, 2006). Moreover, carnivorous arthropods can use HIPV to locate their victims (Dicke, 1999). A well‐studied example of the role of volatiles in plant defence is the tritrophic interaction among lima bean (*Phaseolus limensis*, plant), spider mites (*Tetranychus urticae*, herbivore) and predatory mites (*Phytoseiulus persimilis*, carnivore). After damage by *T. urticae*,* P. limensis* leaves release a complex volatile blend containing homoterpenes that play a crucial role in plant indirect defence to attract the predators of herbivores (de Boer *et al*., 2004). When the emission of homoterpenes was inhibited by the terpenoid pathway inhibitor fosmidomycin, reduced attraction of the predatory mite *P. persimilis* was observed (Mumm *et al*., 2008).

Although monoterpenes and sesquiterpenes are two major classes of HIPV, homoterpenes are the most often reported volatiles (Dicke, 1994; Pateraki *et al*., 2015). Two unusual acyclic homoterpenes with irregular carbon skeletons, a C11 homoterpene, DMNT, and a C16 homoterpene, TMTT, are not only constituents of flower fragrances (Loughrin *et al*., 1994) but are also released from many plant species after herbivore damage. Lima bean and thale cress (*Arabidopsis thaliana*) plants release homoterpenes to attract predatory mites when attacked by spider mites (Lee *et al*., 2010; Mumm *et al*., 2008). Rice (*Oryza sativa*) plants produce homoterpenes highly attractive to females of *Cotesia chilonis* after attack by the striped rice stem borer *Chilo suppressalis* (Li *et al*., 2017). *Campoletis sonorensis* and *Cotesia marginiventris* also respond to homoterpenes released by *Spodoptera littoralis*‐infested cotton (*Gossypium herbaceum*) or maize (*Zea mays*) plants (Gouinguené *et al*., 2005). However, these homoterpenes are generally unable to be detected in undamaged and mechanically damaged foliage (Paré and Tumlinson, 1997).

In *A. thaliana*, AtCYP82G1 can convert (*E,E*)‐geranyllinalool to TMTT (Lee *et al*., 2010). Additionally, TMTT emitted from *Pieris rapae*‐infested *A. thaliana* can attract the natural enemy *Cotesia rubecula* (Kappers *et al*., 2005; Van Poecke *et al*., 2002). DMNT and TMTT are also induced and released from herbivore‐attacked cotton plants (Loughrin *et al*., 1994; Loughrini *et al*., 1995; McCall *et al*., 1994; Rodriguez‐Saona *et al*., 2002). However, the CYP genes involved in homoterpene biosynthesis in *Gossypium hirsutum* remain unclear, and the influence of DMNT and TMTT on the natural enemies of target insect pests in *G. hirsutum* is rarely reported. Encouragingly, the well‐characterized ancestry of cotton and the availability of full genome sequences for *G. hirsutum* provide a useful framework to explore plant indirect defence at the genomic level (Paterson *et al*., 2012).

In this work, emission of DMNT and TMTT from herbivore‐injured cotton plants was confirmed by gas chromatography–mass spectrometry (GC‐MS). Enzymatic activities of putative recombinant CYPs in *Saccharomyces cerevisiae* were investigated by solid‐phase microextraction (SPME) coupled with GC‐MS, and two CYP genes regulating homoterpene metabolism were newly identified. Subsequently, quantitative real‐time PCR (qPCR) was performed to determine the transcript abundance of these genes in different plant organs. Furthermore, the effects of DMNT and TMTT on parasitic wasps of cotton major pests were evaluated by electroantennogram (EAG) and behavioural response assays under laboratory conditions.

## Results

### Emission of DMNT and TMTT from herbivore‐injured *G. hirsutum*

**Figure 1:**
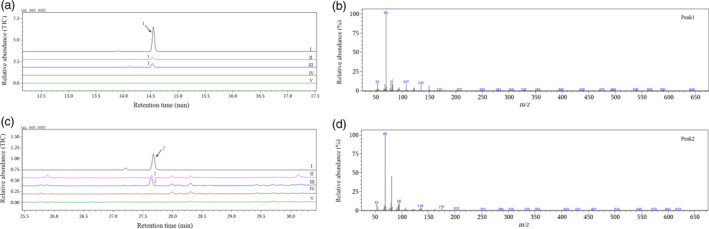
GC‐MS analysis of DMNT and TMTT emitted from cotton plants. (a) I, gas chromatogram of authentic DMNT; II, volatiles from cotton plants infested with *H. armigera*; III, volatiles from cotton plants infested with *A. lucorum*; IV, volatiles from control cotton plants; V, volatiles from an empty glass jar; (b) mass spectrum of peak 1; (c) I, gas chromatogram of authentic TMTT; II‐V are the same as described in (a) II–V; (d) mass spectrum of peak 2. 1, DMNT; 2, TMTT.

GC‐MS analysis of headspace volatile compounds from herbivore‐injured cotton plants showed that both DMNT and TMTT were emitted from *Helicoverpa armigera*‐ and *Apolygus lucorum*‐damaged cotton plants, while neither DMNT nor TMTT was released from herbivore‐free control plants (Figure 1). The amounts of DMNT and TMTT produced were calculated by comparing the peak area ratio to an internal standard (Table 1). These results confirmed the existence of homoterpene biosynthetic pathways in *G. hirsutum* and the participation of particular genes in homoterpene metabolism.

**Table 1:** DMNT and TMTT collected from control and damaged cotton plants

Compound	Undamaged plants	*H. armigera*‐damaged plants	*A. lucorum*‐damaged plants
DMNT	ND	680 ± 65	825 ± 44
TMTT	ND	432 ± 51	928 ± 152

Amounts (means ± SD) measured in ng/h. ND, not detected.

### Identification of candidate CYP genes

**Figure 2:**
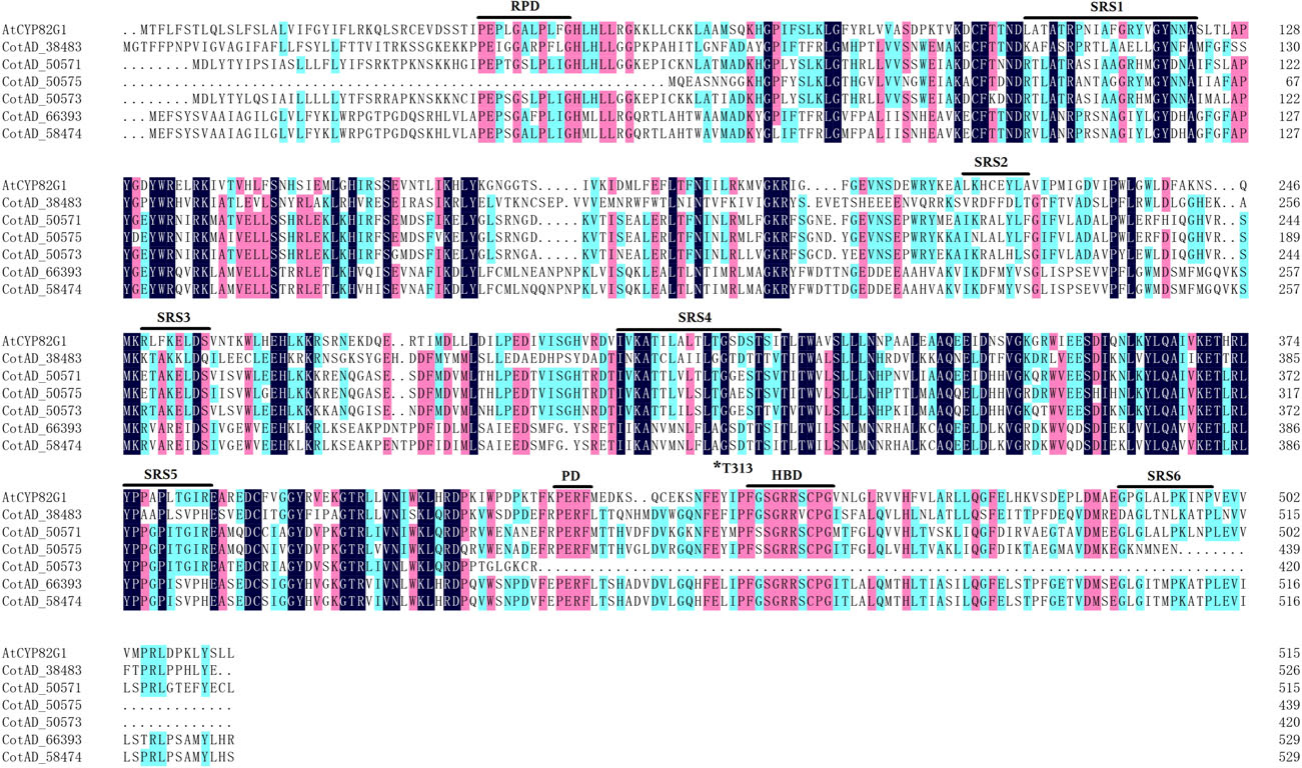
Alignment of deduced amino acid sequences of candidate CYPs with AtCYP82G1. PRD, proline‐rich domain; PD, PERF domain; HBD, heme‐binding domain. SRS1‐6 indicates substrate recognition sites as predicted for AtCYP82G1. Residues with an asterisk represent the key substrate‐interacting residues identified in AtCYP82G1.

According to the proposed biosynthetic pathways of homoterpenes in plants, P450 enzymes are assumed to catalyse the final degradation step. It has been reported that AtCYP82G1 is responsible for TMTT formation in *Arabidopsis*, so the AtCYP82G1 amino acid sequence was employed as a template to blast protein sequences of the *G. hirsutum* genome. Given the existence of conserved domains among P450s, together with the enzyme binding site of the characterized AtCYP82G1 enzyme (Figure 2), CotAD_38483, CotAD_50571, CotAD_50575, CotAD_66393 and CotAD_58474 were selected as candidate genes.

### Catalytic functions of putative CYPs *in vitro*

**Figure 3:**
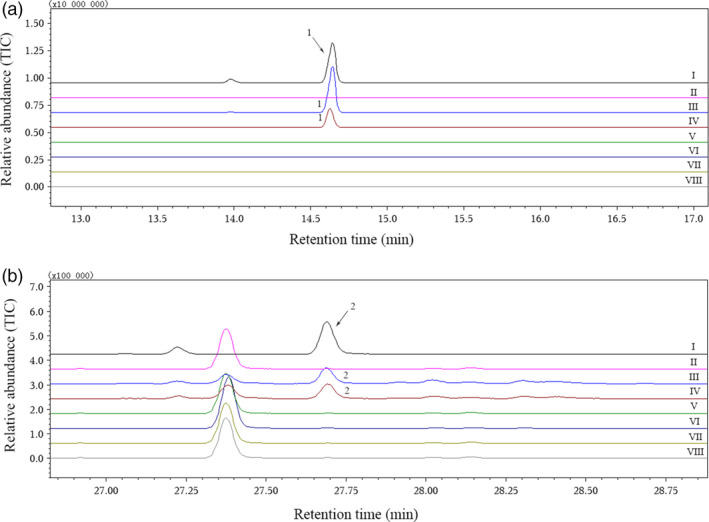
GC‐MS analysis of DMNT and TMTT produced by recombinant proteins expressed in *S. cerevisiae* with C_15_ or C_20_ substrates. (a) I, gas chromatogram of authentic DMNT; II–VIII, volatiles produced by recombinant proteins CotAD_38483, CotAD_50571‐1, CotAD_50571‐2, CotAD_50575, CotAD_66393, CotAD_58474 and empty vector pYES2, respectively, with (*E*)‐nerolidol; (b) I, gas chromatogram of authentic TMTT; II–VIII, volatiles produced by recombinant proteins CotAD_38483, CotAD_50571‐1, CotAD_50571‐2, CotAD_50575, CotAD_66393, CotAD_58474 and empty vector pYES2, respectively, with (*E,E*)‐geranyllinalool. 1, DMNT; 2, TMTT.

**Figure 4:**
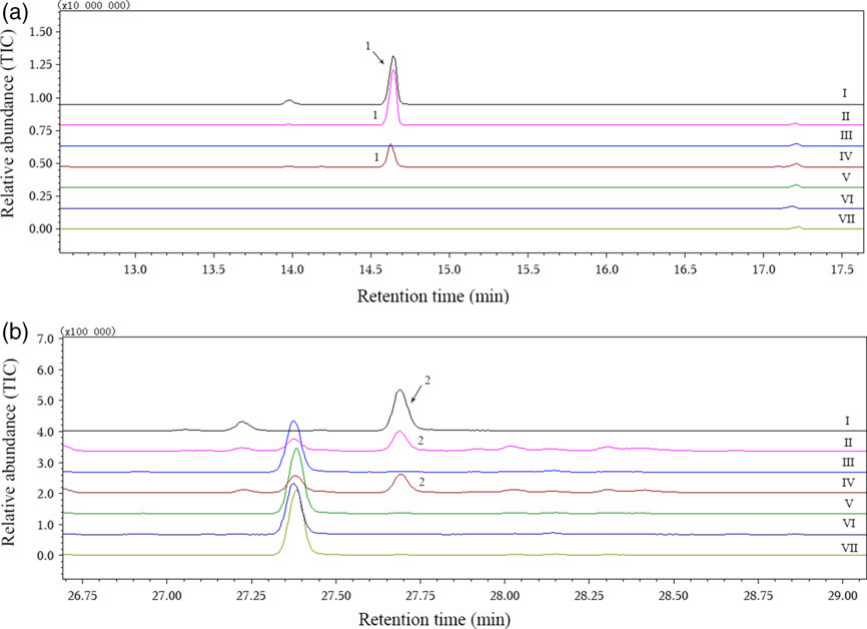
Effect of CPR on the enzymatic activity of recombinant CotAD_50571 proteins. (a) I, gas chromatogram of authentic DMNT; II–VII, volatiles produced by recombinant CotAD_50571‐1 protein co‐expressed with CPR, CotAD_50571‐1 without CPR, CotAD_50571‐2 with CPR, CotAD_50571‐2 without CPR, empty vector pYES2 with CPR and empty vector pYES2 without CPR, respectively, when (*E*)‐nerolidol was used as substrate; (b) I, gas chromatogram of authentic TMTT; II–VII, volatiles produced by recombinant CotAD_50571‐1 protein co‐expressed with CPR, CotAD_50571‐1 without CPR, CotAD_50571‐2 with CPR, CotAD_50571‐2 without CPR, empty vector pYES2 with CPR and empty vector pYES2 without CPR, respectively, when (*E,E*)‐geranyllinalool was used as substrate. 1, DMNT; 2, TMTT.

When we amplified the candidate nucleotide sequences with gene‐specific cloning primers (Table 2), two highly homologous gene sequences (CotAD_50571‐1 and CotAD_50571‐2) were obtained from CotAD_50571. Finally, six recombinant plasmids were constructed from CotAD_38483, CotAD_50571, CotAD_50575, CotAD_66393 and CotAD_58474, and the enzymatic activities of all the recombinant proteins were analysed. Using the suggestions of mass spectra libraries (NIST and Department of Chemical Ecology, Gothenburg University, Sweden) together with the GC retention times and mass spectra of authentic standards, it was found that only CotAD_50571 had the ability to convert (*E*)‐nerolidol to DMNT or (*E,E*)‐geranyllinalool to TMTT (Figure 3). However, the recombinant CotAD_50571 proteins without CPR could not degrade the substrates to the corresponding homoterpenes (Figure 4).

**Table 2:** Primers used in this study

Primer name	Sequence (5′–3′)
*Gene cloning*	
CotAD_38483‐forward	ATGGGAACTTTCTTTCCAAACC
CotAD_38483‐reverse	TTATTCATACAGATGAGGGGGAAG
CotAD_50571‐forward	ATGGATCTTTACACTTACATTCCATC
CotAD_50571‐reverse	TTAAAGGCATTCGTAAAACTCAGT
CotAD_50575‐forward	ATGCAAGAAGCTAGCAACAAT
CotAD_50575‐reverse	CTAGTTTTCATTCATATTTTTACCTT
CotAD_66393‐forward	ATGGAATTCTCATATTCTGTAGCG
CotAD_66393‐reverse	TTAGGTCCTGTGCAGATACATTG
CotAD_58474‐forward	ATGGAATTCTCATATTCTGTAGCG
CotAD_58474‐reverse	TTAAGTGCTGTGCAGATACATTG
*qPCR*	
GhCYP82L1‐forward	ATTCTCTGGTAACGAGTT
GhCYP82L1‐reverse	AACACTGATTACTGAGTC
GhCYP82L2‐forward	ATTCTCCGGTAACGATTA
GhCYP82L2‐reverse	AACATTGATTATTGAGTCGA
Actin‐forward	ATCCTCCGTCTTGACCTTG
Actin‐reverse	TGTCCGTCAGGCAACTCAT

### Homology analysis of target CYPs

**Figure 5:**
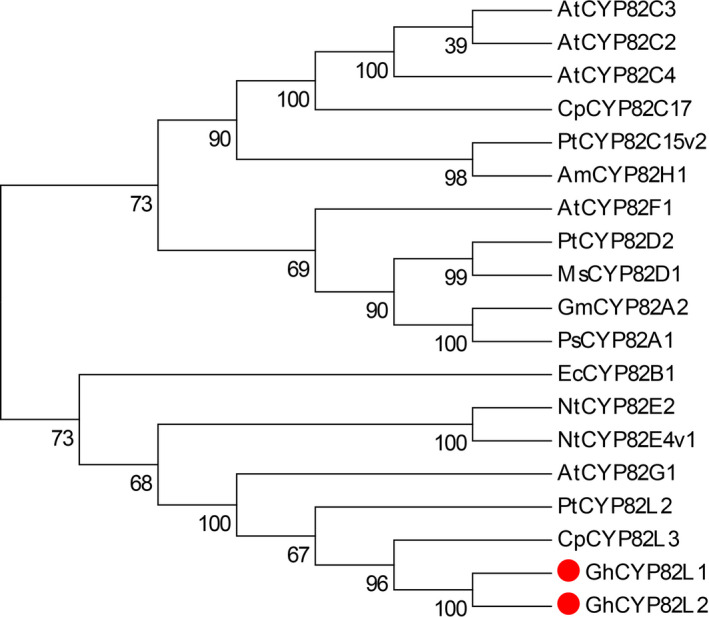
Phylogenetic relationships of P450s of the plant CYP 82 family. Multiple alignments were performed with Clustal W, and the tree was generated with MEGA 6.0. The two sequences marked with dots were used for homoterpene biosynthesis in *G. hirsutum*. Am, *Ammi majus*; At, *Arabidopsis thaliana*; Cp, *Carica papaya*; Ec, *Eschscholzia californica*; Gm, *Glycine max*; Ms, *Medicago sativa*; Nt, *Nicotiana tabacum*; Ps, *Pisum sativum*; Pt, *Populus trichocarpa*.

From the phylogenetic tree of P450s in the plant CYP82 family, the two target CYPs showed the highest identity with CpCYP82L3 (Figure 5), with values of 61.83% and 59.92%, respectively. In addition, the identity between the two target sequences was 95.34%. According to the nomenclature and phylogenetic classification of cytochrome P450s, the two target genes are alleles and classified into the CYP82L family and were deposited in GenBank with the accession numbers KY247144 (*GhCYP82L1*) and KY247145 (*GhCYP82L2*).

### Transcript abundance of *CYP82L*s in herbivore‐damaged and control *G. hirsutum*

**Figure 6:**
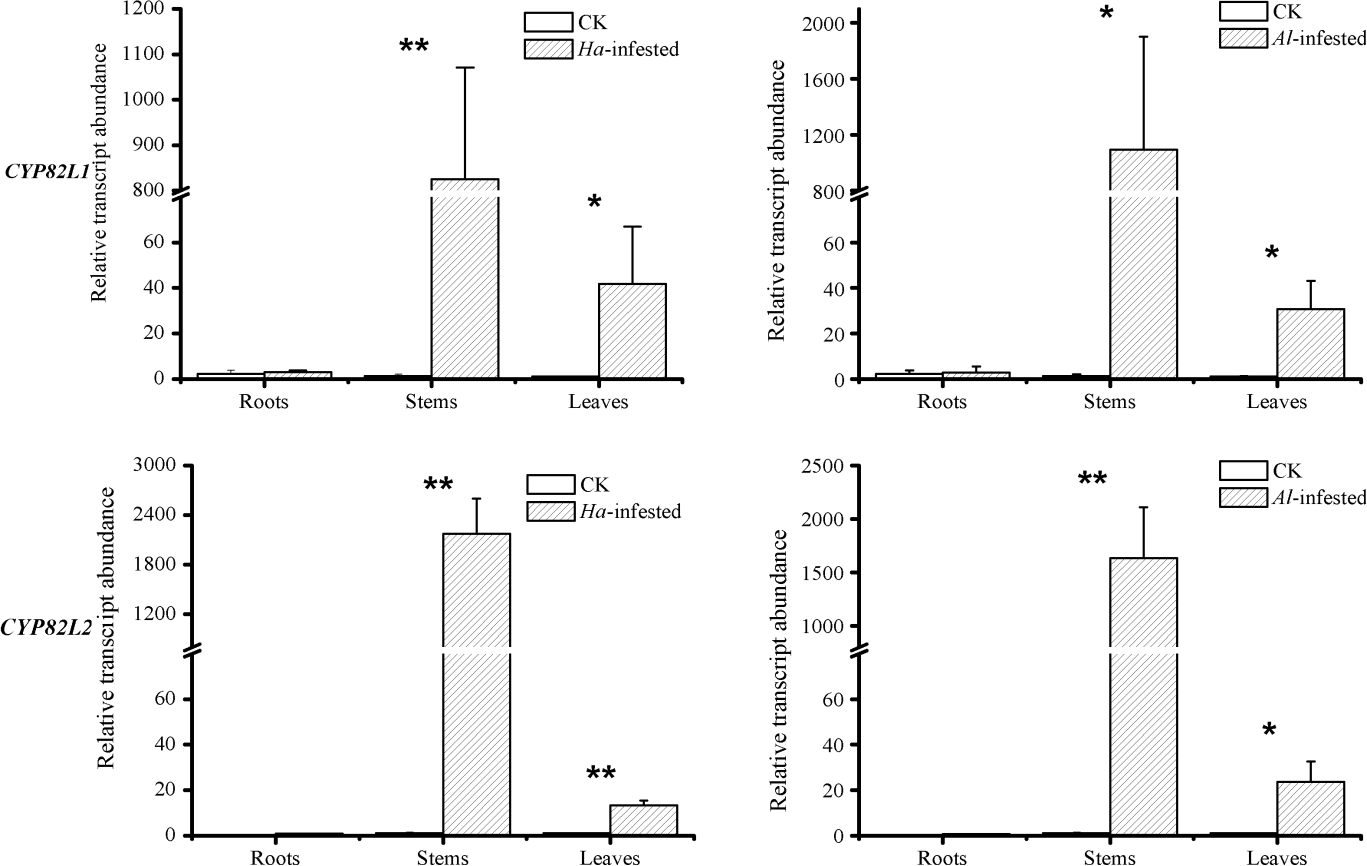
Transcript abundance of *CYP82L* genes in different organs of *G. hirsutum*. Data represent the means ± SE. Asterisks indicate significant differences between treatments (**P < 0.01, *P < 0.05).

To investigate the target gene expression in herbivore‐damaged and control plants, qPCR measurements were conducted to evaluate the transcript levels of *CYP82L*s in leaves, stems and roots of different treatments (Figure 6). *CYP82L1* and *CYP82L2* showed similar expression patterns, with the highest transcript levels in stems, moderate transcript accumulation in leaves and trace accumulation in roots. The two *CYP82L*s showed significantly up‐regulated expression in stems and leaves of herbivore‐attacked plants in comparison with undamaged control plants (*H. armigera*‐infested vs. control treatments: *P*_CYP82L1, leaves_ = 0.02, *P*_CYP82L1, stem_ < 0.01, *P*_CYP82L1, roots_ = 0.80, *P*_CYP82L2, leaves_ < 0.01, *P*_CYP82L2, stem_ < 0.01, *P*_CYP82L2, roots_ = 0.19; *A. lucorum*‐infested vs. control treatments: *P*_CYP82L1, leaves_ = 0.04, *P*_CYP82L1, stem_ < 0.05, *P*_CYP82L1, roots_ = 0.87, *P*_CYP82L2, leaves_ = 0.01, *P*_CYP82L2, stem_ < 0.01, *P*_CYP82L2, roots_ = 0.11).

### EAG responses of parasitic wasps to homoterpenes

**Figure 7:**
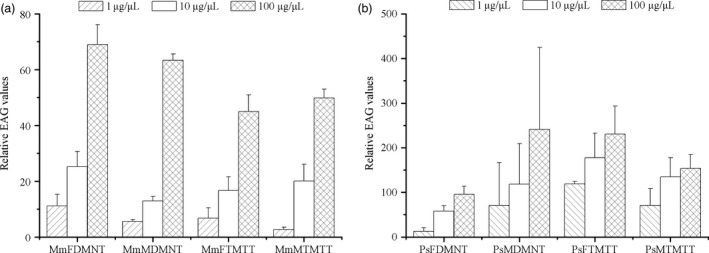
EAG responses of *M*. *mediator* and *P. spretus* to DMNT and TMTT. (a) MmF, females of *M*. *mediator*; MmM, males of *M*. *mediator*; (b) PsF, females of *P. spretus*; PsM, males of *P. spretus*.

The EAG responses of *Microplitis mediator* and *Peristenus spretus* to DMNT or TMTT generally increased as concentrations increased (Figure 7). However, there were no significant differences between males and females of the tested wasps to homoterpenes (for *M. mediator* male vs. female to DMNT: 1 μg/μL, *P* = 0.36; 10 μg/μL, *P* = 0.19; 100 μg/μL, *P* = 0.41; for *M. mediator* male vs. female to TMTT: 1 μg/μL, *P* = 0.47; 10 μg/μL, *P* = 0.76; 100 μg/μL, *P* = 0.65; for *P. spretus* male vs. female to DMNT: 1 μg/μL, *P* = 0.60; 10 μg/μL, *P* = 0.62; 100 μg/μL, *P* = 0.52; for *P. spretus* male vs. female to TMTT: 1 μg/μL, *P* = 0.33; 10 μg/μL, *P* = 0.35; 100 μg/μL, *P* = 0.28).

### Behavioural responses of parasitoids to homoterpenes

**Figure 8:**
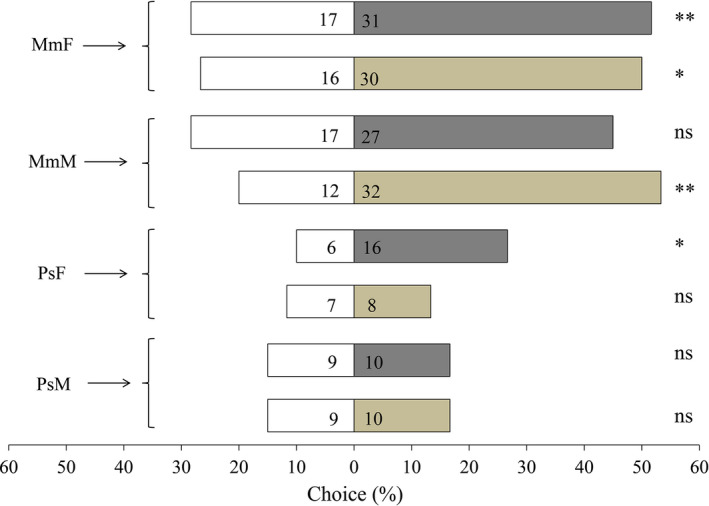
Behavioural responses of *M*. *mediator* and *P. spretus* to DMNT and TMTT. MmF, females of *M*. *mediator*; MmM, males of *M*. *mediator*; PsF, females of *P. spretus*; PsM, males of *P. spretus*. The percentage of wasps that chose mineral oil (white bars) versus DMNT (grey bars) or mineral oil (white bars) versus TMTT (tawny bars) are shown in figure. The numbers on each bar represent the number of wasps that made a choice. Sixty wasps in total were tested in each treatment. Asterisks indicate a significant difference with a choice test: **P <* *0.01, *P <* *0.05, and ns indicates no significant difference.

The behavioural responses of *M. mediator* and *P. spretus* to homoterpenes were studied in a Y‐tube olfactometer. The results showed that *M. mediator* females were significantly attracted by DMNT (χ^2^ = 7.41, df = 1, *P *<* *0.01) and TMTT (χ^2^ = 4.26, df = 1, *P* = 0.04) compared to the mineral oil control, while *M*. *mediator* males showed higher preference only for TMTT (χ^2^ = 9.09, df = 1, *P *<* *0.01), and there was no significant preference of *M. mediator* males to DMNT or mineral oil (χ^2^ = 2.27, df = 1, *P* = 0.13). In *P. spretus*, there was a significant attraction of females to DMNT (χ^2^ = 4.55, df = 1, *P* = 0.03), while no statistically significant preference of males for DMNT or mineral oil was observed (χ^2^ = 0.00, df = 1, *P* = 1.00). Neither males nor females of *P. spretus* had significant preference for TMTT (PsM, χ^2^ = 0.05, df = 1, *P* = 0.82; PsF, χ^2^ = 0.07, df = 1, *P* = 0.80; Figure 8).

## Discussion

### Homoterpenes were induced by herbivore damage in *G. hirsutum*

Emissions of DMNT and TMTT were observed only from *H. armigera*‐ or *A. lucorum*‐damaged *G. hirsutum* in this study. They were also reported to be released only from *G. hirsutum* infested with leaf‐chewing caterpillar *Spodoptera exigua* or piercing‐sucking bug *Lygus hesperus* compared to herbivore‐free plants (Loughrin *et al*., 1994; Williams *et al*., 2005). The result indicated that DMNT and TMTT were emitted exclusively from herbivore‐damaged *G. hirsutum* plants, and the observed homoterpene metabolism was induced by herbivore damage.

### Two CYP82L genes were involved in DMNT and TMTT biosynthesis of *G. hirsutum*

Among all the tested recombinant proteins, only GhCYP82L1 and GhCYP82L2 could catalyse conversion of (*E*)‐nerolidol to DMNT or (*E*,*E*)‐geranyllinalool to TMTT. It was previously reported that AtCYP82G1 could degrade (*E,E*)‐geranyllinalool to TMTT in *A. thaliana* (Lee *et al*., 2010). ZmCYP92C5 was capable of converting (*E*)‐nerolidol to DMNT by oxidative degradation, and ZmCYP92C6 was specific for the conversion of (*E,E*)‐geranyllinalool to TMTT in *Z. mays* (Richter *et al*., 2016). Homoterpene formation in *Z. mays* does not depend on CYP82‐type P450s, as this family is absent in monocots (Tholl *et al*., 2011), whereas ZmCYP92C5 and ZmCYP92C6 were classified into the CYP92 family within the stress‐responsive CYP71 clan, which included the CYP82 and CYP92 families (Richter *et al*., 2016). The GhCYP82Ls and AtCYP82G1 belonged to the same CYP82 family in dicots. The results also suggested that the GhCYP82Ls in *G. hirsutum* could share similar catalytic functions with AtCYP82G1 in *A. thaliana*.

### CPR was necessary for the catalytic action of GhCYP82Ls

Enzyme activity assays revealed that the recombinant proteins without CPR could not degrade (*E*)‐nerolidol to DMNT or (*E,E*)‐geranyllinalool to TMTT. When the target genes were co‐expressed with CPR from *Cucumis sativus*, the recombinant protein could convert substrates to DMNT or TMTT successfully, which indicated that GhCYP82Ls associated with CPR could achieve optimal enzyme activities. It was reported that co‐expression with CPR was essential for CYP to perform catalytic activities (Pompon *et al*., 1996). In *Arabidopsis*, CYP71A13 without CPR and NADPH could not convert indole‐3‐acetaldoxime to indole‐3‐acetonitrile (Klein *et al*., 2013). In poplar, CYP71B40v3 and CYP71B41v2 catalysed the dehydration of aldoximes to nitriles without further oxidation, independent of added CPR (Irmisch *et al*., 2014).

### Transcript abundance of *CYP82L*s also suggested that the expression of *CYP82L*s in *G. hirsutum* was induced by herbivore damage

The expression of *GhCYP82L*s in *G. hirsutum* was significantly up‐regulated after herbivore damage, especially in leaves and stems. These results were consistent with those of *ZmCYP92C*s in *Z. mays*,* PtCYP79D*s in *Populus trichocarpa* and *AtCYP82G1* in *A. thaliana*, which were strongly up‐regulated in herbivore‐damaged plants compared to undamaged controls (Irmisch *et al*., 2014; Lee *et al*., 2010; Richter *et al*., 2016). The highly expressed *GhCYP82L*s contributed to the formation of homoterpenes in herbivore‐damaged plants. Leaves damaged by herbivores could cause systematic defence in cotton plants, which would induce the expression of *GhCYP82L*s in stems. The reasons caused higher expression level of *GhCYP82L*s in stems should be further investigated in details. Moreover, tissue‐specific expression patterns of CYP genes might be helpful in enhancing plant fitness upon herbivore attack. For example, *AtCYP76C1* exclusively expressed in flowers could reduce floral attraction and favour protection against visiting insect pests (Boachon *et al*., 2015), and *AtCYP705A1*, as a root‐specific gene of *A. thaliana*, was expressed to defend against the root rot oomycete pathogen *Pythium irregulare* (Sohrabi *et al*., 2015). High expression of *GhCYP82L*s in the aerial parts of *G. hirsutum* was presumed to enhance the emission of homoterpenes to attract herbivore enemies.

### The potential roles of DMNT and TMTT in attraction of herbivore enemies

Electroantennogram and behavioural studies accompanied by proper identification of semiochemicals not only increase our knowledge of insect chemical communication but also help in making appropriate plant protection strategies (Khan *et al*., 2010). It was reported that DMNT and TMTT were able to attract natural enemies of arthropod herbivores when released from damaged foliage (Tholl *et al*., 2011). DMNT and TMTT, with other induced volatiles from *T. urticae*‐infested *Phaseolus lunatus* leaves, could affect the foraging behaviour of *P. persimilis*, while neither DMNT nor TMTT as a single synthetic compound was attractive to *P. persimilis*. Moreover, volatiles induced by *S. exigua* had significant attractiveness to *P. persimilis* after TMTT was added (de Boer *et al*., 2004). Severely reduced emission of DMNT or TMTT when *P. lunatus* was treated with fosmidomycin could lead to a reduced attraction to predatory mites (Mumm *et al*., 2008). Therefore, specific compounds from complex herbivore‐induced volatiles could play an important role in the behavioural choice of natural enemies of herbivorous arthropods. In this study, EAG assays confirmed that both DMNT and TMTT could be perceived by male or female parasitoids as attractants. Y‐tube assays further showed that females of both wasp species responded positively to DMNT, and males as well as females of *M. mediator* were attracted by TMTT.

Volatile blends were promising for application in integrated pest management strategies that employ volatiles attracting herbivore enemies in the so‐called push–pull systems (Khan *et al.,* 2008). It was also reported that manipulation of TMTT was an ideal platform for pest control via the attraction of generalist and specialist predators in different manners (Brillada *et al*., 2013). The roles of DMNT and TMTT in attracting parasitoids of herbivores have spurred growing interest in improving natural plant defence via the genetic engineering of DMNT and TMTT formation. *C. chilonis* were more attracted to rice plants with overexpression of TPS3 and TPS4 genes of *P. lunatus*, which released more DMNT and TMTT than wild‐type rice plants (Li *et al*., 2017). Transgenic *Lotus japonicus* plants with the TPS2 gene of *P. lunatus* produced TMTT, and the specialist *P. persimilis* was strongly attracted to herbivore‐damaged *L. japonicas* expressing this gene (Brillada *et al*., 2013). The identified *GhCYP82L*s in *G. hirsutum* could also be used as target genes for modification by transgenic techniques to manipulate DMNT or TMTT formation in plant self‐defence, which would provide new strategies for pest management.

## Experimental procedures

### Plant and insect material

Cotton seeds (*G. hirsutum* cv. CCRI12) were sown in plastic pots (16 cm i.d. × 14 cm height) with a 2 : 1 mixture of soil and vermiculite (Yinong Nursery Substrates Co. Ltd, Shandong, China) and grown in a glasshouse (29 ± 4 °C, 40 ± 10% RH, 16L : 8D photoperiod). Plants with 6–7 fully expanded leaves were used for all the experiments (Huang *et al*., 2015).

Larvae of *H. armigera* were reared on an artificial diet under conditions of 27 ± 2 °C, 75 ± 10% RH and 14L : 10D photoperiod (Huang *et al*., 2015). Second‐instar larvae were used for further experiments. Nymphs of *A. lucorum* feeding on green beans (*Phaseolus vulgaris*) were cultivated in climatic chambers at 29 ± 1 °C, 60 ± 5% RH and 14L : 10D photoperiod (An *et al*., 2016). Three‐day‐old *A. lucorum* adults were employed for the following assays. *M. mediator*, a parasitoid of *H. armigera* larvae, was reared in a plexiglass cage (30 × 30 × 25 cm) in a growth chamber (28 ± 1 °C, 60 ± 10% RH, 16L : 8D photoperiod). Newly emerged wasps were maintained with 10% honey solution (Wang *et al*., 2015). *P. spretus*, a parasitoid of *A. lucorum*, was maintained with 10% honey solution as described above under conditions of 25 ± 1 °C, 65 ± 5% RH and 14L : 10D photoperiod (Luo *et al*., 2015). Three‐day‐old adult wasps were prepared for the experiments.

### Plant treatments

Two *A. lucorum* adults or *H. armigera* larvae were placed on each leaf of a pair of cotton plants. Plants without herbivore damage under the same conditions were used as controls. After 24 h, one plant of the group was immediately used for collection of herbivore‐induced plant volatiles. The roots, stems and leaves of the other were harvested, and the collected samples were immediately frozen in liquid nitrogen for PCR assay. For each treatment, three biological replicates were conducted.

### Collection and identification of volatiles

One pot containing one herbivore‐injured or control plant was put into a glass jar (25 cm in diameter × 60 cm in height), and the container was tightly sealed with metal camps on the lid. Air, purified by passage through an activated charcoal filter, was actively pumped through the container at a flow rate of 1500 mL/min with a vacuum pump. Volatiles emitted from herbivore‐injured or control plants were collected in an 8‐mm‐diameter glass tube with 50 mg of 60/80 mesh Tenax‐TA (Shanghai ANPEL Scientific Instrument Company, Shanghai, China) for 8 h (Huang *et al*., 2015). The collected compounds were then extracted with 300 μL of hexane (Fisher, Fairlawn, NJ), to which 8.6 μg of ethyl caprate (Sigma‐Aldrich, Oakville, Canada) was added as an internal standard for quantitative analysis. A 1‐μL aliquot of the extracted sample was splitlessly injected in a GCMS‐QP2010SE (Shimadzu, Japan) equipped with an Rtx‐5 MS dimethylpolysiloxane column (30 m × 0.25 mm × 0.25 μm, Agilent Technologies, CA). Purified helium was used as carrier gas at a constant flow rate of 0.8 mL/min. The injector, transfer line and ion source temperatures were set at 250, 280 and 250 °C, respectively. The GC oven temperature was initially maintained at 40 °C for 1 min and then increased to 190 °C at a rate of 5 °C/min, held for 5 min and finally increased to 250 °C at a rate of 10 °C/min and held for 5 min. In addition, MS was scanned at a 1‐kV detector voltage over 50–650 atomic mass units. Tentative identifications of DMNT and TMTT were made by comparison of mass spectra (a) with mass spectra libraries (NIST and Department of Chemical Ecology, Göteborg University, Sweden) and (b) with mass spectra and retention times of authentic samples obtained from Fluka, Sigma (http://www.sigmaaldrich.com; Huang *et al.,* 2013; Pickett *et al*., 2003).

### Identification of candidate CYP genes

To identify sequences of putative CYPs that regulate DMNT and TMTT formation, a BLASTP search with 10^−5^ as the cut‐off *e*‐value was performed against amino acid sequences of *G. hirsutum* using a sequence of one characterized CYP monooxygenase, *AtCYP82G1* (At3g25180), from *Arabidopsis*. In addition, all the differentially expressed CYPs from previous transcriptome data of *G. hirsutum* infested with *A. lucorum* (data not shown) were also screened. To identify candidate CYPs, the filtered CYP sequences were aligned with representative sequence AtCYP82G1 using the domains conserved among P450s (Xu *et al.,* 2015; Rupasinghe and Schuler, 2006), including the proline‐rich domain (PRD, PxxxxxxP), PERF domain (PD, PERF), heme‐binding domain (HBD, FxxGxxxCxG) and substrate recognition sites (SRS).

### Heterologous expression in *S. cerevisiae*

The complete open‐reading frames (ORFs) of putative *CYP* genes were amplified with gene‐cloning specific primers (Table 1). These sequences were then cloned into the pYES2 vector as *Kpn*I‐*Xba*I or *Hin*dIII*‐Xba*I fragments co‐expressing with CPR of *C. sativus*. The recombinant constructs were transformed into the INVSc1 strain, and transformed cells were selected on SC‐U selection medium. Expression of target genes was performed in the yeast strain INVSc1 according to the manufacturer's instructions (Invitrogen, Carlsbad, CA). Briefly, a single colony containing the recombinant construct was inoculated in 10 mL SC‐U liquid medium containing 2% raffinose at 30 °C in a shaking incubator at 280 r.p.m. until the OD_600_ of the culture reached 1.0; then, the cells were cultured in induction medium containing 2% galactose to express the recombinant protein under the conditions mentioned above for 48 h.

### CYP activity assays

Enzyme activity assays of CYPs were performed in 20‐mL PTFE/Silicon Septa screw cap glass vials (Agilent Technologies). The reaction system, containing 5 mL resuspended culture harbouring recombinant proteins pelleted from 50 mL induction medium and 10 μM (*E*)‐nerolidol or (*E,E*)‐geranyllinalool, was incubated at 30 °C on a temperature‐controlled tray for 4 h. The reaction was terminated by adding HCl to a final concentration of 0.05 M (Lee *et al*., 2010). An SPME (SAAB‐57330U, Bellefonte) fibre coated with 100 μm polydimethylsiloxane/divinylbenzene (PDMS/DVB) was rapidly inserted into the headspace of the vial to capture the reaction products for 1 h at 30 °C. Yeast cells containing empty pYES2 plasmids were used as a control. After absorption, the SPME fibre was directly inserted into the GC injector, and the catalytic products were analysed by SPME‐GC‐MS as described above.

### RNA extraction and qPCR

Total RNA was extracted from collected tissue samples of *G. hirsutum* using the EASYspin Plant RNA kit (Aidlab Biotech, Beijing, China). The quantity of RNA obtained was determined using a Nanodrop ND 2000 (Nanodrop Technologies, Wilmington, DE), and cDNA was synthesized using the SuperScript™ III Reverse Transcriptase Kit (Invitrogen, Carlsbad, CA) according to the manufacturer's instructions. The qPCR measurement was conducted on an ABI7500 PCR System (Applied Biosystems, Carlsbad, CA). Actin (GenBank accession number: AY305733) was used as reference gene. Gene‐specific primers (Table 2) for two target genes, and reference gene were designed using Beacon Designer 7.9 (Premier Biosoft, Palo Alto). All samples were assayed in 20 μL reaction systems using the Talent qPCR PreMix kit (Tiangen Biotech Co. Ltd, Beijing, China) according to the manufacturer's instruction. The PCR cycling parameters were as follows: 95 °C for 3 min, followed by 40 cycles of 95 °C for 5 s and 60 °C for 32 s. Three technical replicates were done for each sample.

### Electroantennogram assay

EAG was used to record the electroantennal responses of two parasitic wasps, *M. mediator* and *P. spretus*, to DMNT and TMTT (ChangZhou NingLu Biological Technology Co., Ltd, Jiangsu, China). Concentrations of chemicals tested were 1, 10 and 100 μg/μL. For each compound, 1‐nonanal at a concentration of 10 μg/μL was used as a reference and liquid paraffin as a control. Filter paper strips (4 mm × 30 mm) loaded with 10 μL of each compound were inserted into a Pasteur pipette. An activated carbon‐filtered airflow at 300 mL/min was passed through the Pasteur pipette, which was placed 5 mm away from the antenna (Zhou *et al*., 2014). Each compound was tested with an interval of at least 30 s on three female and three male adult antennae separately.

### Behavioural response trial

Insect behavioural responses to DMNT and TMTT were evaluated using a Y‐tube olfactometer, which consisted of a 20‐cm‐long central tube and two 20‐cm‐long lateral arms with an interior diameter of 3 cm. The two branch tubes were attached to separate odour‐source flasks. Ten microlitres of each tested chemical (100 μg/μL) was dripped onto a filter paper strip, which was then put into one odour‐source flask. Liquid paraffin in the other flask was used as control (Williams *et al*., 2010). Three‐day‐old parasitoids were individually released at the base of the central arm of the Y‐tube and observed for 5 min. If a parasitoid did not make a choice during this period, it was removed and recorded as no choice. Parasitoids that travelled 2/3 of the distance into the terminal arms and stayed there at least 5 s were recorded as having made a choice. After five runs, the position of the arms was reversed to avoid position bias. The Y‐tube was changed after every 10 individuals tested (Bruce *et al*., 2008). Sixty insects of each species were used in one treatment. All behavioural assays were conducted between 8:00 AM and 12:00 AM.

### Data analysis

The comparative 2^−ΔΔCT^ method (Livak and Schmittgen, 2001) was used to calculate the relative transcript levels of *GhCYP82L1* and *GhCYP82L2* in organs of *G. hirsutum*. In addition, a paired‐sample *t*‐test was employed to examine significant differences in transcript levels of *CYP82L*s between controls and treatment groups (Irmisch *et al*., 2012). Relative EAG values of each parasitoid to volatiles were calculated as described previously (Yang *et al*., 2016). The paired‐sample *t*‐test was also employed to examine significant differences in EAG responses between sexes of tested wasps. In the behaviour trial, we performed a chi‐square analysis with a 50 : 50 distribution to determine the preference of wasps between odour sources and controls. For this analysis, we included only parasitoids that had made a choice. All data were analysed using SAS 9.2 (SAS Institute Inc. Cary, NC).

## Conflict of interest

The authors declare no conflict of interests.
